# Supplementary material for: Genomics of human and chicken Salmonella isolates in Senegal: Broilers as a source of antimicrobial resistance and potentially invasive nontyphoidal salmonellosis infections
Source: PLoS One. 2022 Mar 24;17(3):e0266025. doi: 10.1371/journal.pone.0266025 (PMC8947133; doi:10.1371/journal.pone.0266025)
Supplement: Attachment — Submitted filename: Review Comments_PONE-D-21-24873.docx [file pone.0266025.s003.docx]

**Title of Manuscript (**PONE-D-21-24873): Genomic comparison of clinical human to chicken meat Salmonella isolates in Senegal: broilers as a source of antimicrobial resistance and potentially invasive nontyphoidal salmonellosis infections

1^st^ Author: Yakhya Dieye, PhD

**Review Comments**

1. Throughout the manuscript you have used ‘antibiotics’ and ‘antimicrobials’ interchangeably. Best to use one consistently – I think ‘antimicrobials’ would be more suitable for this type of manuscript that compares clinical (human) and animal isolates

2. Page 74-75: Please revise sentence to read Food animals (poultry, cattle and pigs) and their products (including eggs, milk and pork) constitute ….

3. Line 371-372: You could add to that statement on these lines a further statement to read, ‘or where such regulations exist, they are often not enforced’

**Overall verdict:** The paper is well written and offers analysis of AMR from a somewhat one-health approach (has human and animal samples). Future similar analyses will be strengthened by incorporating environmental and plant samples in an expanded dataset. I recommend it for publication once the above minor corrections have been addressed.
